# Attosecond intra-valence band dynamics and resonant-photoemission delays in W(110)

**DOI:** 10.1038/s41467-021-23650-7

**Published:** 2021-06-07

**Authors:** S. Heinrich, T. Saule, M. Högner, Y. Cui, V. S. Yakovlev, I. Pupeza, U. Kleineberg

**Affiliations:** 1 Max-Planck-Institut für Quantenoptik (MPQ), 85748 Garching, Germany; 2 Ludwig-Maximilians-Universität München (LMU), 85748 Garching, Germany; 3 Department of Physics, University of Connecticut (UConn), Storrs, CT 06269 USA

**Keywords:** High-harmonic generation, Attosecond science, Electronic properties and materials

## Abstract

Time-resolved photoelectron spectroscopy with attosecond precision provides new insights into the photoelectric effect and gives information about the timing of photoemission from different electronic states within the electronic band structure of solids. Electron transport, scattering phenomena and electron-electron correlation effects can be observed on attosecond time scales by timing photoemission from valence band states against that from core states. However, accessing intraband effects was so far particularly challenging due to the simultaneous requirements on energy, momentum and time resolution. Here we report on an experiment utilizing intracavity generated attosecond pulse trains to meet these demands at high flux and high photon energies to measure intraband delays between *sp-* and *d-*band states in the valence band photoemission from tungsten and investigate final-state effects in resonant photoemission.

## Abstract (web summary)

Accessing intraband dynamics is challenging due to simultaneous requirements on energy, momentum and time resolution. Here, the authors measure intraband delays between *sp*- and *d*-band electronic states in the valence band photoemission from W(110) using intracavity generated attosecond pulse trains.

## Introduction

Many important physical properties of solids such as spin magnetism or superconductivity are determined by their electronic valence band. Measurements of the dynamics of valence-band electrons on ultrafast sub-femtosecond timescales reveal important insight into electron–electron correlation^1,2^, dynamic hole screening^3^, and other multi-electron effects in condensed-matter physics that are still not well understood.

Angle-resolved photoemission spectroscopy (ARPES) grants experimental access to the band-structure dispersion *E*(**k**), where the in-band electron momentum ${k}_{\parallel }=\hslash \ast \,\sin \,\alpha \ast \sqrt{2{m}_{e}{E}_{kin}}$ is conserved upon photoemission and can thus be deduced from the kinetic energy *E*_kin_ and the emission angle *α*^4^. Static ARPES measurements have reached energy resolutions below 1 meV^5,6^, whereas time-resolved ARPES intrinsically requires a larger spectral bandwidth such as several tens to hundreds of meV for the femtosecond regime^7–9^. Until very recently, attosecond time-resolved ARPES measurements were not possible because of the stringent and seemingly contradicting requirements on the electron energy resolution (∼100 meV) and requested time resolution (<100 asec).

**Fig. 1:**
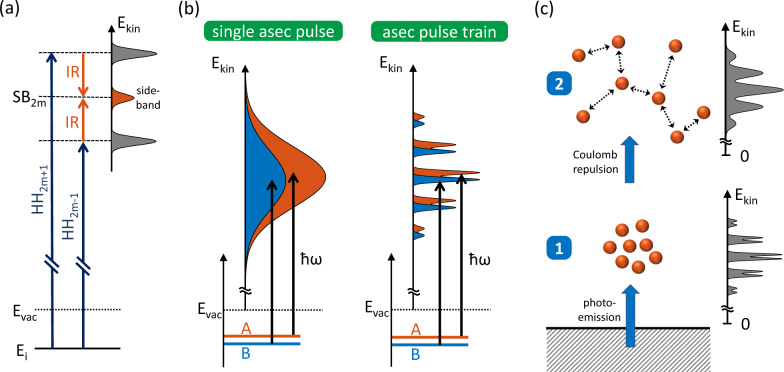
RABBITT sideband principle and energy resolution in attosecond PES experiments. **a** An IR-probe field gives rise to sidebands between separated high-harmonic peaks in a photoelectron spectrum. Each sideband SB_2m_ can be reached by absorption of an XUV photon of the two neighboring high harmonics HH_2m±1_ and the absorption/emission of an IR photon. Because of interference between the two possible paths, the sideband intensity oscillates as a function of the pump–probe delay and gives access to the photoemission timing. **b** Photoelectron spectra from two discrete electronic states A and B excited with a single isolated attosecond pulse (left) and a train of attosecond pulses (right) with the same spectral envelope. The broad continuous spectrum of the isolated pulse makes the photoelectrons from the closely neighbored states largely overlap. In contrast, the narrow and well-separated high harmonics of a pulse train enable a distinction between the photoemission from the two initial states. **c** Schematic representation of space-charge broadening: photoelectrons are released by an attosecond pulse train from a two-state system as in **a**. The repulsive electrostatic force between the photoelectrons leads to space-charge broadening and smears out the fine structure of the photoelectron spectrum.

There are two well-established techniques in attosecond photoelectron spectroscopy (PES): attosecond streaking, which utilizes isolated attosecond pulses^10,11^, and the reconstruction of attosecond beating by interference of two-photon transitions (RABBITT)^12,13^, which employs attosecond pulse trains (APTs). In both approaches, photoelectrons are excited by attosecond pump pulses in the extreme ultraviolet (XUV) and their moment of emission is mapped to their final kinetic energy by the electronic field of typically infrared (IR) probe pulses. In a RABBITT spectrogram, this leads to sidebands (SBs) whose intensity depends on the pump–probe delay (Fig. 1a). Usually, the probe pulse duration is about 5 fs for attosecond streaking and a few tens of femtoseconds for RABBITT experiments. The quantitative agreement between attosecond streaking and RABBITT measurements is well established^14,15^.

Attosecond pulses intrinsically require a large spectral bandwidth from 1.8 eV^16^ to several tens of eV^17^ to support their short duration. However, a train of attosecond pulses in the time domain exhibits a comb-like substructure in the spectrum. These well-separated high harmonics in APTs typically have a spectral width of a few 100 meV. The combination of a broad spectral envelope with narrow harmonics enables experiments with attosecond pulses and energy resolution well below 1 eV (Fig. 1b). This characteristic is indispensable for distinguishing between different initial or final states in solid band structures, especially when combined with angular resolution^18,19^.

However, there is another effect that impairs high energy resolution in attosecond PES experiments on solids: all photoelectrons excited by a single laser pulse are inherently released within very short time and therefore interact with each other. Consequently, space-charge effects lead to shifts and broadening of the photoelectron spectrum (Fig. 1c). Depending on the specific application, this limits the maximum acceptable number of photoelectrons per pulse.

High pulse repetition rates are the obvious solution to this issue: they allow for a high average photoelectron flux with only few released electrons per pulse. Single-pass high-harmonic generation (HHG) systems are typically limited to pulse repetition rates below 100 kHz because of the necessary pulse energies for efficient HHG. Enhancement-cavity-based HHG, however, combines high pulse energies with high repetition rates and is therefore well-suited for (quasi) space-charge free attosecond PES^20^. Thanks to the 18.4 MHz repetition rate, the high average flux of our HHG source translates to only around 500 emitted photoelectrons per laser pulse. Previous investigations have shown that space-charge effects at our parameters do not interfere noticeably with the high energy resolution enabled by the narrow high harmonics^21^.

Although remarkable progress towards the absolute measurement of photoemission timing has been made, it cannot be measured directly either with RABBITT^22,23^ or streaking^24^. In practice, the timing between the IR and XUV pulse cannot be determined with the necessary precision, e.g., due to uncertainties of the interferometric delay and the exact shape of the electric field of the probe pulse. Therefore, measuring photoemission delays ultimately always comes down to referencing photoemission delays from different states or even different materials with each other. Thus, it is common to compare photoemission time delays from different initial states of the same sample with each other, to extract relative photoemission delays within a single measurement^25–28^. Attosecond streaking experiments on solid samples often reference valence-band photoemission delays with those of photoelectrons emitted from deeply bound core states with binding energies of several tens of eV^24,25,29^.

So far, such deeply bound electronic states were not accessible in RABBITT measurements, because it was challenging to obtain the necessary flux of XUV photons with energies in the range of 50–100 eV with many-cycle, high-peak power laser pulses. As the probability of strong-field ionization is proportional to pulse duration, while too much plasma is detrimental for phase matching, HHG with such pulses is less efficient than that with few-cycle pulses, which are used for attosecond streaking. With femtosecond enhancement cavities having reached a maturity that allows for applications in attosecond science^20^, the high peak power enhancement and repetition rate of our cavity-based HHG setup now allows us, for the first time, to address these electronic core states in solids with APTs, thus combining high photon energies (so far reserved to attosecond streaking) with the high energy resolution of separated individual harmonics at high photon flux.

In this study, we present the first attosecond PES experiment, which overcomes the necessity for a tradeoff between high energy resolution and both high photon energies and high photoelectron flux by means of cavity-enhanced HHG at high flux and high repetition rate. By this means, the influence of both initial and final states on the timing of photoemission is investigated.

## Results

### Resonant photoemission delay at 62 eV electron kinetic energy

**Fig. 2:**
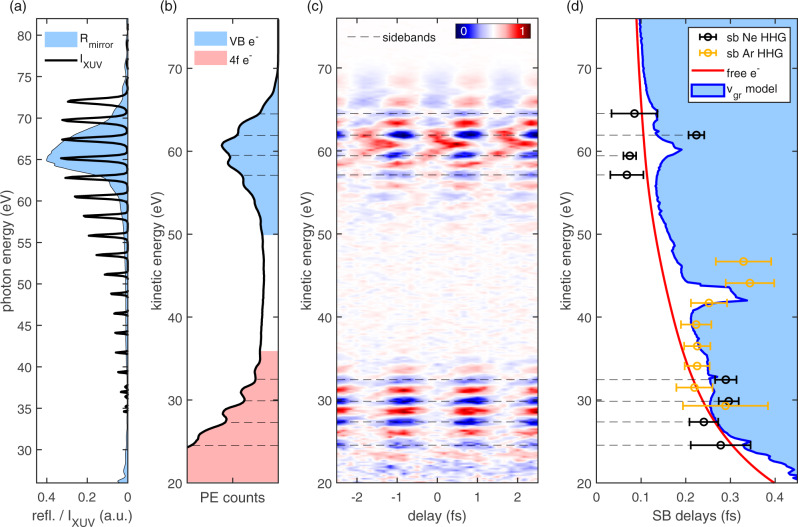
Time-resolved photoelectron spectroscopy from tungsten 4*f* and valence band. **a** XUV spectrum generated in neon (black line) and XUV mirror reflectivity (blue filled area). The XUV spectrum is cut off at 72 eV by a 300 nm Al foil filter. **b** Static photoelectron spectrum with photoelectrons emitted from the tungsten valence band (blue filled area) and the deeper-bound 4*f* state (red filled area). The comb-like appearance of the excitation spectrum is imprinted in the photoelectron spectrum. Sideband positions are indicated by dashed lines (likewise in **c** and **d**). **c** RABBITT spectrogram. Due to depletion, not only the sidebands (predominantly blue) but also the high harmonic peaks (predominantly red) oscillate in intensity. A distinct anomaly in the regularity of the pattern is observed around 62 eV. For better visibility, the displayed spectrogram is background subtracted (see “Methods”). The inset shows the intensities of the sidebands at 59.5 and 61.9 eV (circles), and their fits (lines). **d** Comparison of sideband delays with the electron transport time to the crystal surface. The blue area shows transport times predicted by the final-state group velocities. A classical free-electron propagation results in the red line. Sideband delays extracted from **c** are indicated by black circles. Sideband delays from another RABBITT spectrogram using argon harmonics (see Supplementary Fig. 2a) is indicated by orange circles. The error bars represent the SD over all traces in each dataset (see “Methods”). As only relative photoemission delays are extracted from the spectrograms, an arbitrary delay offset has been added to each dataset to match the calculated delays.

For high-photon-energy attosecond PES experiments at multi-MHz repetition rate, we generated high harmonics in neon and spectrally filtered them with a molybdenum-silicon multilayer mirror, which had a full width at half maximum bandwidth of 5 eV centered at 65 eV photon energy (Fig. 2a). Although the light source provides photon energies in excess of 120 eV, the energy of ∼65 eV is sufficient to address the 4*f* states in tungsten, while still permitting the use of high-transmission aluminum filters below their 72 eV absorption edge in order to spatially separate IR and XUV (see “Methods”).

The resulting photoelectron spectrum features several peaks between 50 and 70 eV, which correspond to valence-band photoemission from the individual high harmonics (Fig. 2b). Separated by their binding energy of 31.2 eV^30^, another set of photoelectron peaks stemming from the 4*f* electron core state is clearly visible at the low-energy part of the spectrum. An XUV-IR pump–probe PES measurement yields a RABBITT trace (Fig. 2c).

In a RABBITT spectrogram, two-photon transitions give rise to SBs in between the photoelectron peaks originating from the high harmonics (Fig. 1a). As a result of quantum interference between the two possible transitions from its neighboring harmonics, the intensity of *S*_*q*_ of the SB of the order *q* oscillates at twice the laser frequency, *ω*_IR_, as a function of the pump–probe delay *τ*_PP_:^12^

1 $${S}_{q}({\tau }_{{\rm{PP}}}){\propto }\,\cos (2{\omega }_{{\rm{IR}}}({\tau }_{{\rm{PP}}}+{\tau }_{{\rm{XUV}},{{q}}}+{\tau }_{{\rm{PE}}}))+{\rm{const}}.$$

The phase of this oscillation comprises a delay *τ*_XUV,*q*_ = (*ψ*_*q*−1_ *−* *ψ*_*q+*1_)/*2ω*_IR_ introduced by the spectral phases of the two neighboring harmonics *ψ*_*q±*1_ and the photoemission delay *τ*_PE_ introduced by the sample under study. Additional probe field induced delays from continuum–continuum transitions are small compared to the observed delays presented in this work and will be neglected^19,22^.

According to Eq. (1), we fit a sine function to each SB *q* and obtain their relative delay, except for the energy- and SB order-independent constant component *τ*_PP_ (see “Methods”). Although the 4*f*-state SB delays are very similar, there is a clear delay anomaly in the SB around 62 eV in the valence band with respect to the others (see open black circles in Fig. 2d). All but this outlier SB closely follow a classical electron transportation model (red line in Fig. 2d), which explains a notable delay in photoemission between the tungsten 4*f* states and the valence band as reported in previous experiments, based on attosecond streaking^24,25^. However, the striking increase in photoemission delay by 148 ± 40 asec of SB 56, at 62 eV kinetic energy, has not been observed before and cannot be attributed to the XUV spectral phase of the harmonic comb, as it does not show up in the corresponding 4*f* SBs at around 30 eV kinetic energy.

Although photoemission delays between different initial states with different orbital momentum have been observed in bulk^31^ and gas^26,28^, the delay deviation at 62 eV in our experiment is almost by an order of magnitude larger than these and can, thus, not be explained by such an initial-state effect. The tungsten 4*f* core state consists of a spin doublet separated by 2.2 eV^30^, whose peaks overlap in the spectrogram. However, simulations according to a recently demonstrated model^32^ have shown that this has hardly any influence on the observed 4*f* SB delays in our experiment, nor could it explain a resonant delay in the valence band (see Supplementary Note 3).

Having ruled out the influences of initial states and excitation as the reason for the observed discrete increase in photoemission delay at 62 eV, it has to be assumed that this is a final-state effect similar to previously reported findings in other transition metals^18,19^.

In one case, an observed final-state induced photoemission delay was explained by the reflection and interference of the electron wave packet in the solid, which can occur if the de Broglie wavelength matches the crystal layer distance^33^. We find not one but two distinct increases in delay and for neither of them the electron wavelength is in agreement with the interlayer distance of 2.24 Å in a W(110) crystal and therefore rule out this explanation for our findings.

In the three-step model of photoemission, the electron transport time to the bulk surface can be modeled by the quotient of the electron inelastic mean free path (IMFP) and the electron wave packet propagation velocity. Tao et al.^18^ attributed a resonant photoemission delay in nickel to the lifetime of the high-lying final state of an excited electron. In the three-step picture of photoemission, they explain this by an increased mean emission depth of the photoelectrons whose energy-velocity relation is not subject to the crystal potential but follows a free-electron dispersion.

Other publications assume that the electron emission depth follows the smooth energy dependence of the universal IMFP curve for all elements^34^ and relate an observed increase in photoemission delay to a decrease in electron propagation velocity induced by the final band structure^17,19^. We found that our results are well explained by this approach with the quotient of the general IMFP in tungsten^35^ and an electron propagation velocity based on the final band dispersion (see Supplementary Note 1) as displayed in Fig. 2d. For comparison, the resulting delay calculated from the free-electron velocity is also shown.

Both calculations clearly support the experimentally observed relative delay of the core-level photoemission compared to the photoemission from the valence band, leading to a very similar expected delay. It is noteworthy that the actual absolute photoemission delay with respect to the XUV pulse is unknown in this measurement and is chosen such that the relative delays in each measurement fit best to the simulation. By taking the group velocities of the final bands into account, two distinct resonant increases in the photoemission delay can be found at around 43 eV and 60 eV final electron kinetic energy. Their positions agree well with the calculated minima of the final band group delays (see Supplementary Note 1). The measured increase in delay at around 62 eV exceeds the model curve in magnitude by about 50 asec. However, as our model relies on the maximal group velocity at each final energy (see Supplementary Note 1), this discrepancy can be explained by contributions of bands with slower group velocities (blue filled area in Fig. 2d).

To further support the validity of our interpretation, we have also performed another RABBITT measurement at a photon energy of ∼52 eV addressing the second resonance at the kinetic energy of 43 eV. This resonance is also clearly visible in the measured data (orange solid circles Fig. 2d). The observation of two different surges in photoemission delay (at 43 and 62 eV) predicted by our electron transport time calculations in two independent measurements corroborates our model.

**Fig. 3:**
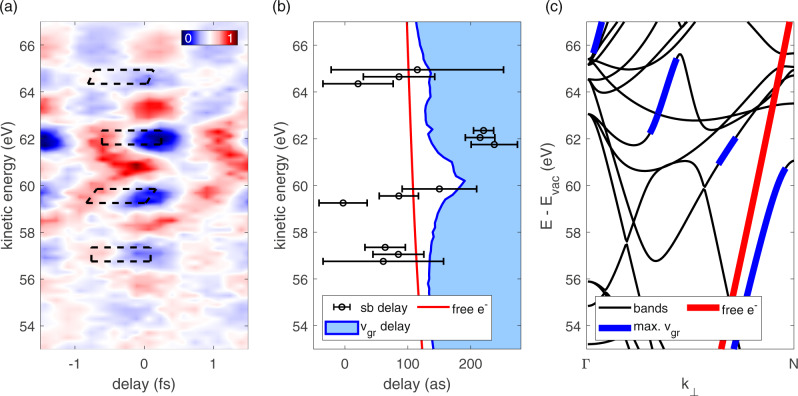
Intra sideband delay evaluation of tungsten valence band photoelectrons around 62 eV. **a** Magnified view of the RABBITT-spectrogram in Fig. 1c around 62 eV. The dashed boxes represent the energy-dependent shape of each sideband. **b** For each sideband, we retrieved the delays at its central, highest, and lowest energies (black circles). Note the asymmetric character of the sideband at 60 eV just below the resonance (see text). Calculated classical electron transport time to the crystal surface is indicated by the red line, the group-velocity-based simulation of transport delays by a blue solid line. As the simulation is based on the maximum group velocities at each final energy, contributions from other bands can lead to higher delays (blue filled area). Error bars represent the SD over all traces in each dataset (see “Methods”). **c** Band energies for electrons photoemitted perpendicularly to the surface (black lines). The classical free-electron dispersion curve is shown in red; the bands with maximum group velocity at each energy are highlighted in blue. The latter are notably more shallow at 60–62 eV, which translates to lower group velocity and therefore higher photoemission delay in **b**.

A closer insight into this resonant delay structure at 62 eV is given by analyzing the energy-dependent shapes of the valence-band SBs as indicated by the dashed boxes in Fig. 3a. Each SB has been analyzed by three linecuts: through its center and through its upper and lower margins. The corresponding delays have been plotted in Fig. 3b. The figure shows that the onset of the resonance already appears in the upper margin of the SB below the actual resonance slightly below 60 eV. The intra-SB delay shift of the highest indicated SB around 65 eV can be attributed to the large error bars at these energies.

This SB shape analysis allows for a more precise experimental evaluation of the resonance shape, magnitude, and position. The calculation of the maximum group velocities perpendicular to the surface in the final bands around the resonance area (58–64 eV) has been derived from band-structure calculations as displayed in Fig. 3c. The classical free-electron parabola is marked in red and the bands contributing to the maximal group velocities are marked in blue. The latter exhibit a shallower behavior at final kinetic energies of 60–62 eV, which is the reason for the lower maximum group velocity in this energy range, and results in longer electron transport times as displayed in Fig. 3b. It is worth noting that the displayed bands are only a representative subset of the contributing part of the Brillouin zone (BZ, see Supplementary Note 1).

### Initial-state photoemission delays within the valence band

Electronic wave functions in crystals are formed (within the tight binding model) by linear combinations of atomic orbitals, commonly referred to as Bloch waves, which are invariant under lattice translation. Although, in general, any electronic state in a solid corresponds to a superposition of an infinite number of atomic states, often just a few atomic orbitals are dominant in a Bloch wave. In transition metals, a Bloch state *ψ*_*l*_*(***k***)* with a band index *l* and a wave vector **k** may largely consist of atomic *d*-orbitals. The corresponding energy band *E(***k***)* is referred to as a *d-*band. Accordingly, energy bands of *sp*-character are formed from atomic orbitals with *s* and *p* orbital momentum.

**Fig. 4:**
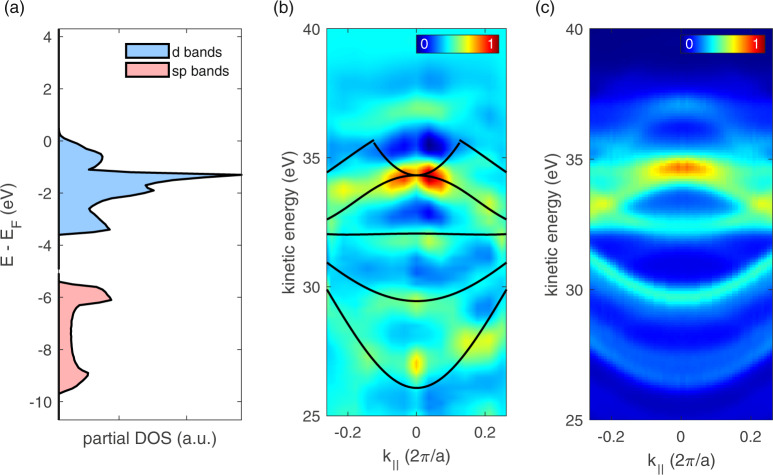
Angle-resolved photoemission spectroscopy. **a** Effective density of states in tungsten as defined by the visible parts of the Brillouin zone at our spectrometer acceptance angle of ±7° around 32 eV kinetic energy. **b** Angle-resolved photoemission spectrogram along the Γ–*P* axis measured in the wide-angle mode of our spectrometer with an angle acceptance of ±15°. Black lines display the occupied bands in the Γ–*P* and *N*–*P* directions. It is noteworthy that this measurement was taken by quasi-monochromatic excitation dominated by one strong harmonic. The displayed bands refer to excitation with this dominating harmonic and are only displayed up to their Fermi level. **c** Simulated angle-resolved photoemission spectrogram for the same geometry and measured XUV spectrum as in **b**.

Although the previous section described final-state effects in the resonant photoemission from the tungsten valence band, in the following experiments we focused on the investigation of these different initial bands within the tungsten valence band. As low photon energies are sufficient for this purpose, we generated high harmonics in argon. As compared to neon, HHG in argon provides a significantly higher XUV flux with energies between 33 and 50 eV. The angular acceptance of ±7° of our electron spectrometer, the final electron kinetic energy (around 32 eV), and the (110) orientation of the crystal determine the observable part of the tungsten valence band BZ. The resulting partial density of states (DOS) over all populated and accessible band parts is displayed in Fig. 4a. It is composed of a sharp dominating peak of *d*-electron states 1.6 eV below the Fermi energy and a broader and weaker contribution of deeper-bound *sp*-electrons with a band gap of around 2 eV between these two components.

To spectrally resolve these different parts of the tungsten valence bands in a RABBITT experiment, it is thus necessary to choose an excitation bandwidth comparable to the width of the band gap that still allows for at least two strong high harmonics for a well-modulated SB signal. Therefore, we opted for an XUV mirror with a bandwidth of 4 eV centered at 41 eV.

To identify the different *sp*- and *d*-contributions in the valence band by their dispersion, we performed ARPES (Fig. 4b). The data reproduce the key features of the band structure with the upwards open parabola-shaped dispersion of the *sp*-band around 30 eV kinetic energy separated from the flat or downwards open dispersion of the *d*-band around 35 eV. The **k**_||_ projection of the band structure along the Γ–*P* and *N*–*P* directions (black solid lines Fig. 4b) coincides well with the experimental data. As the excitation is not monochromatic, the displayed data consist of a superposition of spectrograms for each harmonic. We simulated this by convoluting the **k**_||_-dependent DOS with the utilized XUV spectrum and accounting for the instrumental resolution. The result is displayed in Fig. 4c and is in qualitative agreement with the experimental data shown in Fig. 4b.

**Fig. 5:**
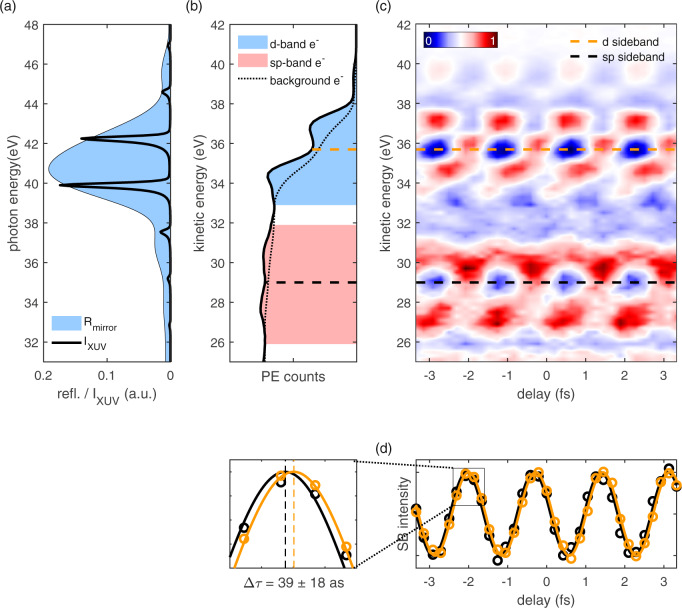
Time-resolved photoelectron spectroscopy in the tungsten valence band. **a** XUV spectrum generated in an argon gas target (black line) and XUV mirror reflectivity curve (blue filled area) with a maximum reflectivity of 19% and a bandwidth of 4 eV. For better comparability with **b** and **c**, their energy plot range is shifted by 6 eV to account for work function and binding energy. The displayed XUV spectrum was measured before the XUV mirror and multiplied with its reflectivity curve. **b** Static photoelectron spectrum featuring several high-harmonic photoelectron peaks without background subtraction. The peaks can be attributed to *sp*- and *d*-bands as indicated by the filled areas (compare Fig. 3a). It is noteworthy that these areas also contain a large amount of secondary electrons (dashed line), which do not contribute to the sideband signal. **c** RABBITT spectrogram featuring two well-separated oscillating sidebands emitted from the *sp*- (black dashed line) and *d*-band (orange dashed line), respectively. For better visibility, a delay-independent background was subtracted (see “Methods”). **d** Linecuts through the sidebands marked in the same colors in **c** and the corresponding sine fits. The *d*-dominated sideband (orange) is delayed by 39 ± 18 asec with respect to the *sp*-band-dominated sideband (black). The error represents the SD over all traces in the dataset (see “Methods”).

Figure 5a displays the generated XUV spectrum and the reflectivity of the utilized silicon–boron carbide multilayer mirror, which filters out two harmonics of about equal intensity, while further harmonics are suppressed. In the corresponding static photoelectron spectrum, the *sp-* and *d*-electron contributions can be well distinguished (Fig. 5b). Time-resolved pump–probe measurements yield a RABBITT spectrogram (Fig. 5c) where the dashed lines indicate the positions of the *sp*- and *d*-band SB. Sine fits to these SBs reveal that photoemission from the *d*-band is delayed by 39 ± 18 asec with respect to the *sp*-bands (Fig. 5d). This corroborates the findings of previous measurements in gas^26,28^ and solids^31^, which generally observed a larger delay for electronic states with a higher orbital momentum.

**Fig. 6:**
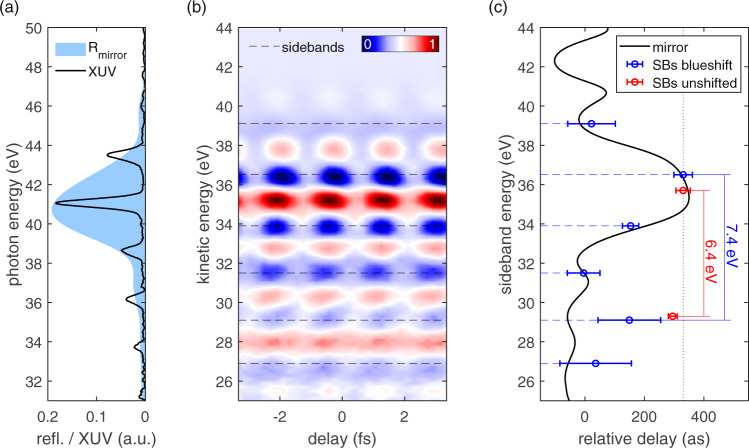
Tungsten valence band RABBITT-measurement with energy shifted high harmonics. **a** XUV spectrum measured after the multilayer XUV mirror and corresponding reflectivity curve. The position of the high harmonics is shifted by 0.8 eV compared to previous measurement (Fig. 4). Hence, the spectrum is dominated by a strong high harmonic at the maximum of the mirror reflectivity curve. The other harmonics are significantly weaker and they have similar heights. For better comparability with **b** and **c**, their energy plot range is shifted by 6 eV to account for work function and binding energy. **b** RABBITT spectrogram taken with the excitation spectrum in **a**. Due to the comparable strengths of most harmonics, many sidebands form (dashed lines), which have similar oscillation amplitudes. **c** The sideband delays extracted from the spectrogram in **b** (blue circles) are in good agreement with the calculated sideband delay introduced by the dispersion of the utilized multilayer mirror (black line). The relative delays extracted from the unshifted RABBITT measurement in Fig. 4c (red circles) strongly differ from the delays obtained from the spectrogram in **b**. The separation between the sidebands at about 29 and 36 eV differs by 1 eV between the unshifted (6.4 eV) and blueshifted spectrogram (7.4 eV). The error bars consist of the SD over all traces in each dataset (see “Methods”) and the errors introduced by the XUV optics (see Supplementary Note 2).

The extracted delay in this measurement is reliable, because it is vastly dominated by only two strong high harmonics and, consequently, only one SB. In this case, the *sp*- and *d*-band valence electrons are excited by the same harmonics and the spectral phase of the XUV does not play a role. However, utilizing the same XUV mirror, another very different situation can be created by shifting the high harmonics in energy. This can be achieved by introducing additional intracavity blueshift by means of moving the HHG gas nozzle closer to the focus, to increase the non-linearity^36^. As the blueshifted spectral components accumulate in the cavity, this effect is significantly stronger than in regular single-pass HHG setups. A spectral shift of about 0.8 eV leads to an XUV spectrum, which is dominated by a single strong harmonic at the peak of the XUV-mirror reflectivity curve (Fig. 6a). Out-of-bandwidth harmonics are suppressed by the mirror but still well visible.

The change from a configuration with two strong harmonics to this spectrum has a strong impact on the measured RABBITT spectrogram (Fig. 6b). As the SB oscillations arise from the interference of the two neighboring harmonics, their modulation depth is determined by the weaker of the two harmonics. Hence, although the central harmonic is by far the strongest, it does not dominate the RABBITT signal to a similar extent and comparably pronounced oscillations can be observed over a relatively broad energy range. This leads to an overlap of lower-order SBs from *d*-bands with photoelectrons originating from the deeper-bound *sp*-bands.

The SB delays extracted from this spectrogram closely follow the delay, which is introduced by the group delay dispersion of the XUV mirror (see Fig. 6c). Hence, the observed photoelectrons (and SBs) originate mainly from the same initial state (i.e., the dominant *d*-band) and about seven different high harmonics such that the SB delays match their spectral phase. A weaker contribution of *sp*-electrons overlaps with *d*-band electrons at lower kinetic energies and leads to a certain deviation from the mirror induced a delay curve for the SBs at about 27 and 29 eV. This situation is very different from the situation with only two strong harmonics displayed in Fig. 5c, where the spectral phase of the XUV has no influence, because both observed SBs stem from the same high harmonics, but different initial states (*sp*- and *d*-band). Consequently, the initial-state induced delay difference between the two SBs (red circles in Fig. 6c) was noticeably smaller than the mirror induced delay difference between the SBs in the blueshifted spectrogram at similar kinetic energies (blue circles in Fig. 6c). The different energy separation between the SBs at around 36 and 29 eV for the unshifted (6.4 eV) and blueshifted spectrogram (7.4 eV), corroborates this interpretation (see Fig. 6c). In the shifted RABBITT trace, this corresponds to three high-harmonic spacings, whereas in the unshifted trace the valence band substructure creates this energy gap.

The possibility of easily shifting SBs to this extent can be a very useful tool for the observation of effects at discrete kinetic energies in future experiments, as well as for distinguishing between initial- and final-state effects.

## Discussion

We have presented the first RABBITT measurement on deeply bound core states in a solid, which is also the first XUV attosecond experiment at MHz repetition rate. This self-referencing measurement on a W(110) crystal features significant resonant delays in photoemission at electron kinetic energy of 62 and 43 eV, which can be attributed to neither initial-state effects nor the XUV spectral phase. Instead, these two resonances clearly resemble the maximum group velocity in the final bands perpendicular to the surface. A more detailed analysis of the SB shapes around the resonances gives a more detailed insight into the position, shape, and magnitude of the resonance, which is linked to the final-state band-structure dispersion. This new technology is thus an extremely powerful tool to investigate the dispersion and group velocities of unoccupied bands on an attosecond timescale. So far, the band structure above the Fermi energy has been hardly accessible in attosecond experiments. This new access allows for the investigation of quasiparticle lifetimes with attosecond precision.

Furthermore, our experiment has shown that different bands within the valence band can be distinguished in a RABBITT spectrogram, which allow for intra-valence band referenced measurements. Angle-resolved photoemission measurements and simulations corroborate these findings and identify the different contributions to stem from *sp*- and *d*-angular orbital-dominated parts of the band structure. An upper limit of 39 ± 18 asec of the *sp*-photoemission delay with respect to the *d*-band photoemission is extracted from the measurement, which can be explained in terms of an orbital-momentum-dependent inner-atomic delay. This intra-valence band analysis is not possible for broadband attosecond pulses, as they are utilized in isolated attosecond pulse streaking experiments and have thus not been seen in these previous streaking experiments.

The unique combination of high flux, high photon energy, and high energy resolution together with angularly resolved detection of our experiment will enable further valuable insights into band-structure-dependent electron dynamics, revealing a new understanding of electron–electron scattering and correlation effects upon photoemission of more complex electronic materials on attosecond timescales.

## Methods

### Cavity-based HHG and PES setup

The laser system consists of a Ti:Sa oscillator seeding a Yb-doped fiber amplification system with actively stabilized carrier-envelope phase^37^. The repetition rate of 18.4 MHz rate guarantees space-charge free experimental conditions^21^, avoids cumulative effects in the HHG gas target^38^, and ensures a temporal detection duty cycle of photoelectrons close to 100%^21^. After amplification, the 1030 nm pulses are spectrally broadened in a multi-pass cell and temporally compressed^39^ to a final pulse duration of 39 fs at 83 W of average power. These pulses are coupled into a passive resonator with an enhancement factor of 35 and XUV photons are generated via HHG in a noble gas target in a focus and, subsequently, output coupled through a hole in a cavity mirror^40^.

Although the setup is optimized for HHG in argon with an output coupled flux of 10^13^ photons per second at up to 60 eV, it can be run with neon without major modifications at significantly higher photon energies of up to 120 eV at the expense of XUV photon flux (10^10^ photons per second). Hence, we can do inter-band-referenced measurements at deeply bound states with neon HHG, while argon HHG is perfectly suited for angular-resolved high-precision studies of valence band dynamics. Due to its low divergence, the XUV beam can be spatially separated from the IR by a 300 nm-thick Al filter in the center of the beam, which is opaque for the fundamental laser light. A two-segment XUV multilayer mirror was used to introduce a variable delay between the IR and XUV components, and focused both beams on a tungsten (110) single crystal. Subsequently, up to 10^6^ photoelectrons per second emitted within a ±7° solid angle are captured by an angle-resolving time-of-flight spectrometer. For wide-angle applications, an additional spectrometer mode with an angle acceptance of ±15° is available and was used for the data presented in Fig. 4b. For more detailed information regarding the setup, please refer to ref. ^21^.

### Tungsten band structure and DOSs

All theoretical data of the electronic structure of tungsten in this work was calculated with the open source software QUANTUM ESPRESSO, which relies on density function theory, plane waves, and pseudopotentials^41^. We assumed a body centered cubic tungsten crystal with a lattice constant of *a* = 3.165 Å^42^ and used a Perdew–Burke–Ernzerhof functional type ultrasoft pseudo-potential. Our crystallographic orientation is (110) such that the photoelectron emission is centered around Γ–*N* axis. At a fixed electron spectrometer angle acceptance *α*, the maximum observable parallel electron momentum *k*_||_ depends on the electron kinetic energy *E*_kin_ according to ${k}_{\parallel }=\hslash \ast \,\sin \,{\rm{\alpha }}\ast \sqrt{2{m}_{{\rm{e}}}{E}_{{\rm{kin}}}}$. At ±7° angle acceptance in the electron kinetic energy range from 30 to 65 eV, this leads to a maximum accessible *k*_||_ of 0.17–0.25 2*π*/*a*. Hence, the part of the BZ, which contributes to our PES measurements, is at a fixed kinetic energy, a cylinder around the emission direction Γ–*N*. Summation over all these states at 32 eV kinetic energy yields the partial DOSs depicted in Fig. 4a. By keeping the *k*_||_ information, we obtained a *k*-space-resolved DOS, which was used to calculate the simulated angular-resolved photoemission spectrogram in Fig. 4c. For consistency, we confirmed that our DOSs over the entire BZ is in very good agreement with literature^43^.

### Spectrogram background subtraction and illustration

As the subtraction of a delay-independent background is not necessary for the SB delay analysis and has absolutely no impact on it, static background subtraction is a purely cosmetic affair. However, for illustration, it is very helpful and we have been using two approaches at it. The suppression of the constant fraction of the spectrogram in the Fourier space is very helpful to emphasize the time dependency of the SB intensities. The drawback of this method is that no difference between oscillating SBs and the oscillating harmonic peaks can be discerned after applying the Fourier filter. In contrast, subtracting a delay-averaged and smoothed photoelectron spectrum visualizes well the SB and harmonic peak positions in the spectrogram, because the harmonic peaks clearly emerge over this kind of background. A linear combination of these two backgrounds guarantees optimum visibility of the relevant features of a RABBITT trace and was subtracted from all displayed spectrograms in this work with individually adjusted suitable weighting. To make the spectrograms more pleasing to the eye, additional points were interpolated between the measured data points for a smoother appearance. As an example, the process from the raw data to the spectrogram displayed in Fig. 6b is illustrated in Supplementary Fig. 4.

### SB delay evaluation

To extract the phases and thereby the delays from the individual SBs, it is helpful to compensate for drifts that occur during the measurement of a RABBITT trace,e.g., due to slow cavity misalignment, which results in a drift of the spectral envelope of the high harmonics. To minimize the influence of this effect, we measured our spectrograms alternating with increasing and decreasing delay. In addition, we removed the lowest non-constant component of the spectrogram in the Fourier space to eliminate such slow drifts during the measurement of a spectrogram without interfering with the SB modulation-related effects.

For the SB delay evaluation, we fit a sine function with a period of 1.718 fs, which corresponds to half the oscillation period of the fundamental laser central wavelength and reproduces the observed oscillation very well. We determine the exact positions of the SBs in the Fourier-transformed RABBITT spectrogram by identifying the kinetic energies with the highest amplitudes at this SB oscillation frequency. For the SB fit, we average over the photoelectron counts in a total kinetic energy range of 500 meV around the central SB energy, which is well below the separation between high harmonics and SBs of 1.2 eV. In the case of the intra-SB analysis in Figs. 3a and 2b, we chose an integration interval of 300 meV for each slice.

The result of the sine fit is the sum of the three delay components in Eq. (1) of the main text: pump–probe delay *τ*_PP_, XUV spectral phase-induced delay *τ*_XUV_, and photoemission delay *τ*_PE_. As *τ*_PP_ is unknown but identical for all kinetic energies and SB orders, we can only extract the delays of the SBs relative to each other with an arbitrary offset that is equal for all SBs and energies, and is chosen such that it matches best the calculated delay curves (in Figs. 2d and 6c). Relative delay components, which depend on SB order and the final or initial energy are preserved. As the attochirp delay *τ*_XUV*,q*_ only depends on the SB order, its non-constant contribution can be distinguished from the one by *τ*_PE_ in measurements with clearly different initial states as for the 4*f* and valence electrons in the neon HHG data in Fig. 2d and the *sp*- and *d*-band electrons in Fig. 5d.

The SB sine-fitting is done for all RABBITT spectrograms taken during a measurement campaign at identical parameters. The total SB delay given in the main text and its figures is the mean value of all the delays extracted from the individual spectrograms and their SD is our error bar. As the signal-to-noise ratio in the neon-HHG data in Figs. 2 and 3 is relatively low, we had to merge a total of 54 measurements into 6 subsets of sums over 9 spectrograms, which was not necessary for the argon HHG datasets for the large bandwidth measurement in Fig. 2d (consisting of 73 traces) and the small bandwidth measurement in Fig. 5c (12 traces) and Fig. 6b (82 traces).

### Crystal preparation

The crystal is mounted in a flag style sample holder, which is held by a frame that is designed to reduce the thermal contact to the vacuum chamber. Hence, the crystal can be heated up to 2200 K by electron impact heating within several seconds and without significantly heating other parts of the vacuum chamber. An actual light-bulb filament is placed about a centimeter behind the tungsten crystal and emits up to 240 mA at a voltage of 1.1 kV when flashing the sample. All parts forming the crystal mount are made of molybdenum to withstand the high temperatures and prevent magnetic stray fields.

Because of our high average laser power due the necessary probe intensity and our high repetition rate, the crystal is permanently exposed to a laser power of >1 W, which heats it up to temperatures of 900 K within several minutes during a cavity lock. This might help to keep the crystal clean longer from adsorption but would be detrimental for most other bulk materials. However, we already plan to implement a different IR beam geometry, which will strongly improve our focus profile and allow for the same probe field intensity at a quarter of the average laser power. If necessary, additional crystal cooling could be installed.

To prevent oxidation of the crystal and the formation of other compounds or an adsorption layer of residual atoms and molecules in the experimental vacuum chamber, it is important to clean the crystal surface regularly. Our crystal preparation routine was based on an available recipe^44^, which consists of several flashes at 1200 K in an oxygen atmosphere to remove carbon followed by a single flash at 2200 K in ultra-high vacuum to remove oxides. The temperature was monitored by a pyrometer and the pressure in our vacuum chamber was between 10^−9^ and 10^−8^ mbar. The procedure was repeated at least every 60 min. We found empirically that the contamination was reduced at a permanent 10^−6^ mbar argon background pressure during the photoelectron measurements and the flashing of the crystal.

### Spectrogram acquisition times and step width

The pump–probe delay step size does not have any influence on the result or the precision of the measurement^45^; hence, we are free to choose a value such that the total delay range spans at least two SB oscillation periods, which are well resolved, which results in an intuitively well-understandable spectrogram. In the case of the neon-dataset in Fig. 2c, we opted for traces of 31 delay steps of 167 asec and a total spectrogram exposure time of 174 s each, for all other displayed measurements with argon high harmonics 33 delay steps of 208 asec.

The entire neon HHG dataset presented in this work (Fig. 2) was taken within a net acquisition time of 145 min within two measurement days. The broadband argon HHG RABBITT dataset presented in Fig. 2d of the main text (orange circles) and Supplementary Fig. 2 was measured within a net acquisition time of 195 min in 1 day and the narrowband RABBITT datasets displayed in Fig. 5c and Fig. 6b of the main text in net 28 and 136 min within 1 day, respectively. Because of the cavity-based HHG setup, the gross acquisition time under typical conditions is roughly a factor of 2 longer, as described in the “Measurement routine” section. Flashing the tungsten crystal will also prolong the actual measurement times. Due to our high repetition rate, it is hence possible to record meaningful spectrograms within as little as 2 min corresponding to 2 × 10^9^ laser shots, which can be highly beneficial for the investigation of quickly degrading samples.

### Measurement routine

Running an enhancement cavity requires to lock the cavity length to the repetition rate of the laser or vice versa, and in our case both these approaches are implemented in a slow and fast loop, respectively^21^. However, vibration-free piezo-actuators with the necessary precision and speed have only a limited range, so that the cavity length drift due to thermal effects must be corrected from time to time by another long-range linear stage. In addition, drifts in the laser system and the cavity alignment will also reduce the overlap of incoming beam with the cavity mode and the position of the cavity mode on the pierced mirror, which both will result in reduced XUV flux. All these minor issues can easily be fixed within little time out of lock. As a consequence, roughly every 5 to 15 min the photoelectron experiments have to be stopped to optimize the cavity. Pump–probe scans have to be concluded within this time frame, because the intracavity IR power and the XUV spectrum can slightly differ between two locks and the XUV-IR interferometer delay can drift. However, due to the high flux of the system, it is no problem to measure one or even several meaningful RABBITT spectrograms within this time, even when generating high harmonics in neon.

## Supplementary information

Supplementary Information

## Data Availability

The data that support the findings of this study are available from the corresponding author upon reasonable request.
