# Supplementary material for: Attosecond intra-valence band dynamics and resonant-photoemission delays in W(110)
Source: Nat Commun. 2021 Jun 7;12:3404. doi: 10.1038/s41467-021-23650-7 (PMC8184802; doi:10.1038/s41467-021-23650-7)
Supplement: Supplementary file 1 — Supplementary Information [file 41467_2021_23650_MOESM1_ESM.pdf]

Supplementary Information

Attosecond intra-valence band dynamics and resonant-photoemission delays in W(110)

S. Heinrich[1,2,†] ✉, T. Saule[1,2,3, †], M. Högner[1,2], Y. Cui[1,2], V. S. Yakovlev[1,2], I. Pupeza[1,2] and U. Kleineberg[1,2]

1 Max-Planck-Institut für Quantenoptik (MPQ), Hans-Kopfermann-Str. 1, 85748 Garching, Germany.
2 Ludwig-Maximilians-Universität München (LMU), Am Coulombwall 1, 85748 Garching, Germany.
3 University of Connecticut (UConn), Dept. of Physics, 196 Auditorium Road, 06269 Storrs, CT, USA.
† authors contributed equally
✉ stephan.heinrich@mpq.mpg.de

Supplementary Note 1: Electron transport time calculation

The total photoemission delay τ_{PE} is composed by the Wigner-delay τ_W due to the absorption of the XUV photon [1] and the transportation time τ_{transp} from the photoelectron to the crystal surface. Because τ_W is small compared to the magnitude of the delays observed in this work and their error bars [2, 3], it will not be included in our simple man's model of the photoemission delay. The electron transport time calculation relies exclusively on the emission depth $\lambda(E_{kin})$ and the velocity v_e of the photoelectron: $\tau_{\mathrm{transp}} = \lambda/v_e$. The mean emission depth is the inelastic mean free path of an electron in a tungsten crystal which depends on the electron kinetic energy and is taken from calculations in [4]. In the case of a classical free-electron-like behavior, where the interaction of the electron with the lattice potential is neglected, the velocity simply is $v_{\mathrm{e}} = \sqrt{2m_{\mathrm{e}}E_{\mathrm{kin}}}$.
Assuming that a photoexcited electron moves in a static periodic crystal potential, group velocities determine how fast electron wavepackets propagate in energy bands. The electron group velocity perpendicular to the surface is given by $v_{\mathrm{gr}} = \frac{1}{\hbar}\frac{dE}{dk_\perp}$ according to the final state's band dispersion $E(k_\perp)$, which is shown in Supplementary Fig. 1a. We calculated these group velocities for each band at 150 discrete values of $k_\perp$ as well as 81 different values of $\mathbf{k}_{||}$ covering the part of the BZ that corresponds to the acceptance angle of our time-of-flight spectrometer. Afterwards, we determined the highest possible group velocity at each electron kinetic energy (see Supplementary Fig. 1b) and calculated the corresponding transport time, which consequently can be considered a lower boundary on the electron transport time.
Since no selection rules or transition probabilities have been considered, this approach is only meant to be an approximation but still coincides well with our experimental data as shown in Fig. 2d in the main text.

Supplementary Note 2: Sideband delays introduced by XUV mirror, filter foil and HHG-process

In Fig. 2d of the main text sideband delays of an Ar-HHG RABBITT experiment are shown and the corresponding spectrogram is displayed in Supplementary Fig. 2a. Due to the fact, that these sidebands are (unlike the others presented in this work) unreferenced to different initial states and due to the very large bandwidth of around 20 eV we had to account for the influence of the group delay dispersion of the employed XUV optics. Hence, to obtain meaningful information from the relative delays between the sidebands in this spectrogram we had to subtract the spectral phase contributions of the utilized molybdenum-silicon XUV multilayer mirror and the 300 nm aluminum filter.

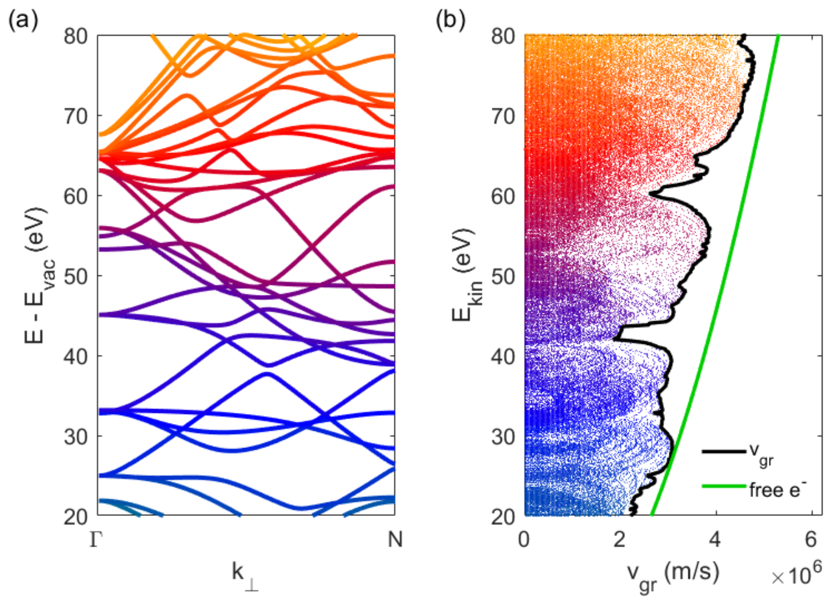

Supplementary Fig. 1 Electron transport time calculation. a) Tungsten electronic band structure along the Γ-N-axis calculated with QUANTUM ESPRESSO [9] **b)** Group velocities (colored dots) derived from the band structure in a) and 80 other k-space axes parallel to the Γ-N direction covering the entire accessible part of the Brillouin zone in $k_{||}$. The extracted maximal group velocity is represented by a solid black line, the classical free electron velocity by a green one.

The spectral phases $\psi_{q\pm1}$ of the two harmonics of the order $q\pm1$ lead to a delay of $\tau_{XUV,q} = (\psi_{q-1} - \psi_{q+1})/2\omega_{IR}$ in the the qth sideband [5]. For a continuous XUV spectral phase $\varphi_{XUV}(\omega)$ and at our IR laser frequency ω_{IR} this can be translated to the following expression:

$$\tau_{XUV}(E_f) = [\varphi_{XUV}(\omega - \omega_{IR}) - \varphi_{XUV}(\omega + \omega_{IR})]/\ 2\omega_{IR} \qquad (1)$$

Here, $\tau_{XUV}(E_f)$ is the continuously calculated sideband delay at the final electron kinetic energy E_f. The latter can be calculated from the photon energy, the material work function Φ and the binding energy as $E_f = \hbar\omega - \Phi - |E_{bind}|$. For a W(110) crystal, the work function is 5.3 eV [6] and 1.5 eV is the binding energy of the most dominant contribution of the tungsten valence band. Therefore, a total of 6.8 eV is subtracted from the photon energy to map it to the final electron kinetic energy.

The spectral phase of the Al filter was calculated from its refractive index taken from [7] and the phase of our in-house made XUV mirrors are reliably calculated from their design [8]. The resulting sideband delays according to Supplementary Eq. 1 are displayed in Supplementary Fig. 2b. Subsequently, the mirror and filter contributions were subtracted from the raw sideband delays (Supplementary Fig. 2b).

The HHG-parameters for simulations in [10] cover a wide parameter range which is very similar to our experimental conditions. An evaluation of the spectral phases of all simulated XUV spectra in this publication yields maximum sideband delays below 2 asec induced by HHG-process related attochirp. Because these contributions of the initial XUV spectral phase can be neglected, the relative sideband delay after subtraction of the influence of mirror and filter can to a great extent be attributed to the target material.

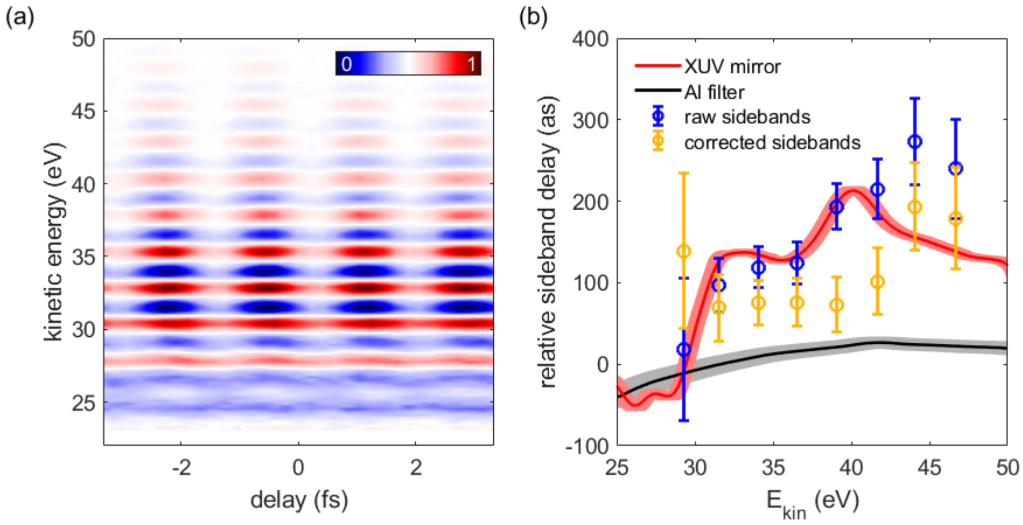

Supplementary Fig. 2 Mirror and aluminum filter influence on broadband RABBITT-measurements. a) RABBITT spectrogram taken with a broadband XUV mirror and high harmonics generated in argon. **b)** The group delay dispersion of the XUV optics directly translates into energy dependent sideband delays which are displayed for the XUV multilayer mirror (red line) and the 300 nm aluminum filter (black line). Both curves are shifted by -6.8 eV in energy to translate photon energy in kinetic energy which accounts for the work function and a representative binding energy of the photoelectrons. The gray and red filled areas represent the errors due to a 10% foil thickness uncertainty, a mirror GDD error of 1000 as^2 and a 500 meV uncertainty in energy due to the spectrometer work function. The measured raw sideband delays we observe in our broadband argon-HHG RABBITT experiment shown in a) (blue circles) are corrected by the mirror and filter delays to obtain the material induced sideband delays (orange circles). The errors induced by mirror and filter are included in the error bars of the corrected data points.

Supplementary Note 3: Tungsten 4f spin doublet sideband reconstruction

The tungsten 4f-electronic state is split into a doublet by spin-orbit-coupling. The substructure consisting of 4f 5/2 and 4f 7/2 level is split by 2.2 eV [11], which corresponds almost exactly to our high harmonic spacing of 2.4 eV and is hence not resolvable with our setup due to the width of the high harmonics and the electron spectrometer resolution. However, an approach to extract information from overlapping RABBITT-interferograms has been proposed and demonstrated recently [12]. In this model the spectrogram is Fourier transformed and only the component oscillating at twice the fundamental laser frequency is kept, as it contains all the necessary sideband delay information (see Supplementary Fig. 3a). This complex-valued vector contains the oscillation amplitude of sideband and high harmonic PES peaks (red line in Supplementary Fig. 3b) as well as their phases (red asterisks in Supplementary Fig. 3c) and thus their delays. In order to model this, three components are necessary: The oscillation amplitudes A_j for each sideband (and harmonic) order j, their phases φ_j and the spectral shape of a single photoelectron peak $p(E)$. The complex $2\omega_{IR}$ component of the spectrogram is then calculated as a sum over the contributions from each sideband and harmonic:

$$Z_{2\omega}(E) = \sum_j A_j * e^{i\varphi_j} * p(E - E_j)$$

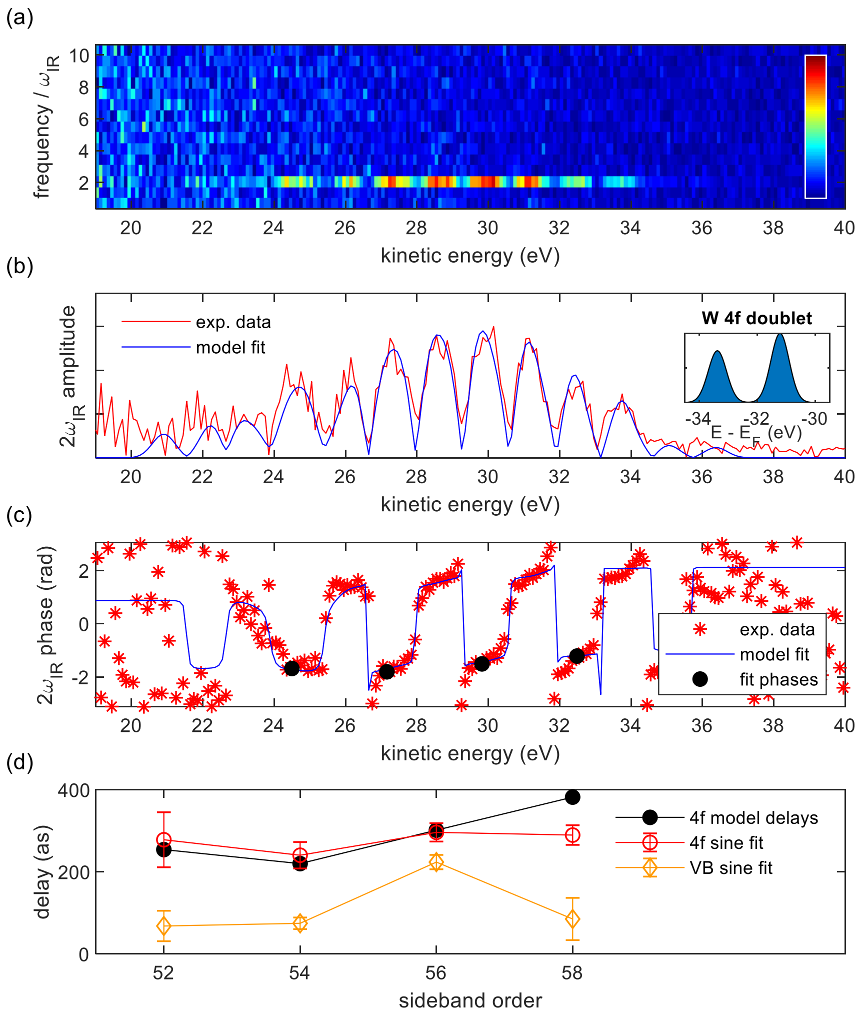

Supplementary Fig. 3 Reconstructing a tungsten 4f-spin-doublet RABBITT spectrogram. a) Absolute value of the Fourier transform of the 4f-part of the RABBITT-spectrogram displayed in Fig. 2c of the main text. The sideband (and depleted high harmonics) contribution oscillating at twice the fundamental laser frequency ω_{IR} clearly shows up in the transformed spectrogram. No significant $4\omega_{IR}$ components can be observed, which corroborates, that we are in a two-photon-transition regime and have no higher order transition contributions. Note, that due to depletion not only the sidebands, but also the high harmonics oscillate at $2\omega_{IR}$. **b)** Amplitudes of the $2\omega_{IR}$ components of the 4f-RABBITT spectrogram (red line) and result of the fit of our model to the experimental data (blue line). The inset displays the line shape of the tungsten 4f doublet, which was used for the reconstruction of the data. **c)** Spectral phases of the $2\omega_{IR}$ components of the 4f-RABBITT spectrogram (red asterisks) and result of the fit of our model to the experimental data (blue line). The phases that the model extracted for the strongest sidebands are marked as black dots. **d)** The resulting 4f sideband delays given by the fit of the model (black dots) are similar to the 4f sideband delays obtained by a simple sine fit (red circles) and do not show a significant increase in delay for sideband 56 as it is the case for the valence band photoelectrons (orange diamonds).

If in the spectrogram the photoelectron contributions of two or more harmonics and sideband overlap, this model will account for and extract the actual phases φ_j of each sideband.
In our case, we insert as spectral shape *p(E)* the 4f double structure which was simply modeled by two Gaussians to fit experimental literature results [11] and folded with a gaussian of 500 meV width to account for high harmonic bandwidth and spectrometer resolution (inset in Supplementary Fig. 3b). The central energy E_j of each sideband/harmonic contains their order *j*, a general energy offset E_0 and the spacing between harmonics and sidebands: $E_j = E_0 + j * \hbar\omega_{IR}$. A total of eleven sidebands and harmonics are considered and the model is then fit to the experimental data and the resulting phase and amplitude are in good agreement with it (blue lines in Supplementary Fig. 3b and 3c).
The extracted sideband phases (black dots in Supplementary Fig. 3c) are converted to delays and are quite similar to the delays extracted from sine-fitting the sidebands. Because the exact shape of the 4f-doublet has not been monochromatically measured in our case, this approach is not very well suited, to gain quantitative results. It is meant to evaluate qualitatively, if the consideration of the 4f doublet structure could yield a comparably high increase in delay in the 56th sideband as observed in the valence band. Because this is clearly not the case (see Supplementary Fig. 3d), it can be excluded, that the large delay of this sideband in the valence band can be attributed to the spectral phase of the high harmonics.

Supplementary Note 4: Spectrogram background subtraction and illustration

The process of the background subtraction and illustration of the spectrograms displayed in this manuscript is described in the Methods section of the main text. In Supplementary Fig. 3 we display a spectrogram as raw unprocessed data and together with the data after background subtraction and interpolation.

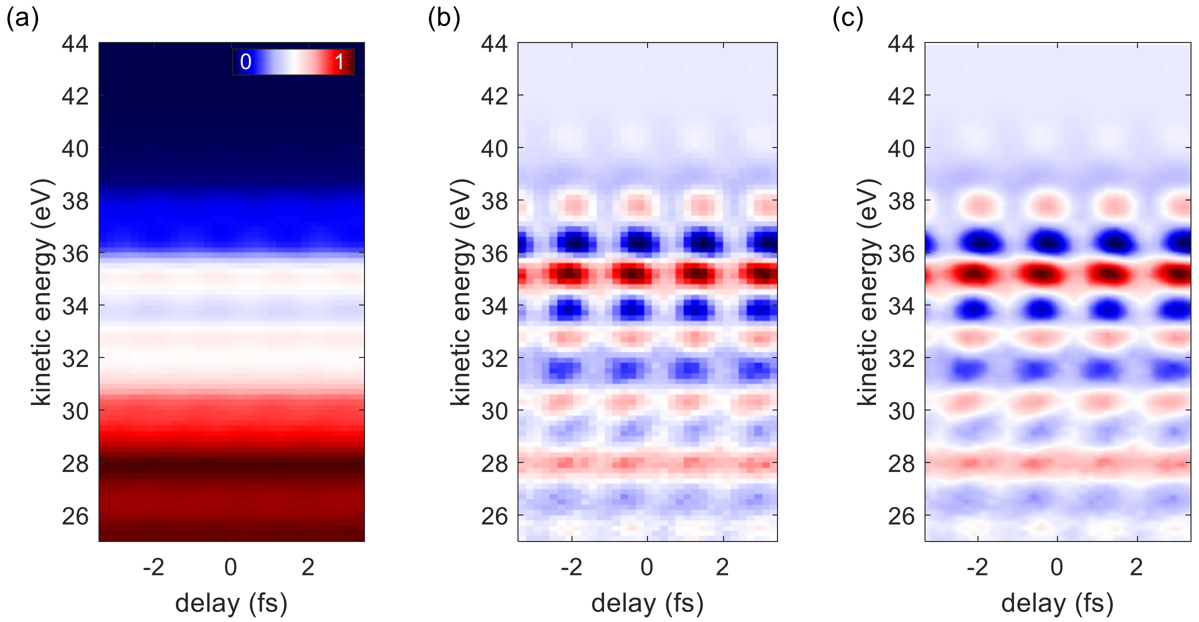

Supplementary Fig. 4 Spectrogram illustration a) Raw measured RABBITT spectrogram of the data displayed in Fig. 5b of the main text. **b)** Spectrogram after subtraction of a delay-independent background. **c)** Spectrogram including additional interpolated points for smoother appearance.

References

[1] Wigner, E. P.Lower Limit for the Energy Derivative of the Scattering Phase Shift. Phys. Rev. **98,** 145 (1955).

[2] Kasmi, L. et al. Effective mass effect in attosecond electron transport. *Optica* **4,** 1492-1497 (2017)

[3] Locher, R. et al. Energy-dependent photoemission delays from noble metal surfaces by attosecond interferometry. *Optica* **2,** 405-410 (2015).

[4] Shinotsuka, H., Tanuma, S., Powell, C. J. &Penn, D. R. Calculations of electron inelastic mean free paths. XII. Data for 42 inorganic compounds over the 50 eV to 200 keV range with the full Penn algorithm. *Surf. Interface Anal.* **51,** 427– 457 (2019).

[5] Paul, P. M. et al. Observation of a train of attosecond pulses from high harmonic generation. *Science* **292,** 1689–1692 (2001).

[6] Strayer, R.W., Mackie, W. & Swanson, L.W. Work function measurements by the field emission retarding potential method. *Surf. Sci.* **34**, 225-248 (1973).

[7] Henke, B. L., Gullikson, E. M. & Davis J. C. *X-ray interactions: photoabsorption, scattering, transmission, and reflection at E=50-30000 eV, Z=1-92. Atom. Data Nucl. Data* **54,** 181-342 (1993).

[8] Guggenmos, A. et al. Aperiodic CrSc multilayer mirrors for attosecond water window pulses, Opt. Express **21**, 21728-21740 (2013)

[9] Giannozzi, P. et al. QUANTUM ESPRESSO: a modular and open-source software project for quantum simulations of materials. *J. Phys.: Condens. Matter* **21,** 395502 (2009).

[10] Högner, M., Saule, T. & Pupeza, I. Efficiency of cavity-enhanced high harmonic generation with geometric output coupling. *J. Phys. B: At. Mol. Opt. Phys.* **52,** 075401 (2019).

[11] Colton, R. & Rabalais, J. Electronic structure of tungsten and some of its borides, carbides, nitrides, and oxides by X-ray electron spectroscopy. *Inorg. Chem.* 1976, **15,** 1, 236–238 (1976).

[12] Jordan, I. & Wörner, H. J. Extracting attosecond delays from spectrally overlapping interferograms. *J. Opt.* **20,** 024013 (2018).
